# Spontaneous splenic rupture associated with apixaban: a case report

**DOI:** 10.1186/s13256-016-1012-6

**Published:** 2016-08-09

**Authors:** Lacy E. Lowry, Jonathan A. Goldner

**Affiliations:** 1 The Commonwealth Medical College, 525 Pine Street, Scranton, Pennsylvania 18509 USA; 2 The Commonwealth Medical College; Pocono Medical Center, 206 East Brown Street, East Stroudsburg, Pennsylvania 18301 USA

**Keywords:** Spleen, Rupture, Apixaban, Abdominal pain, Case report

## Abstract

**Background:**

Spontaneous splenic rupture associated with anticoagulant use is a rare but potentially lethal disorder. Lack of prompt recognition can be associated with poor patient outcomes. The use of novel oral anticoagulants is becoming more common and thus consideration of this disorder while evaluating a patient who presents with abdominal pain while using these agents is extremely important. This is the first reported case of spontaneous splenic rupture associated with apixaban.

**Case presentation:**

We describe the clinical case of an 83-year-old white man who complained of sudden severe abdominal pain 5 days into a hospital stay for acute-on-chronic congestive heart failure and exacerbation of chronic obstructive pulmonary disease. Neither he nor his wife reported any significant trauma for the past 6 months prior to his admission. His medical history included chronic atrial fibrillation treated with medications including apixaban 2.5 mg twice daily. An urgent abdominal computed tomography scan demonstrated a large splenic hematoma and evidence of intraperitoneal bleeding from which he rapidly declined, developing hypovolemic shock. An emergency splenic arteriogram displayed a patent splenic artery and an embolization was successful in stabilizing him. Due to evidence of recurrent bleeding, an exploratory laparotomy and splenectomy was subsequently performed the following day.

**Conclusions:**

The diagnosis of spontaneous splenic rupture is important to consider in a patient using apixaban who presents with abdominal pain and associated signs of hypotension and anemia. For hemodynamically unstable patients, prompt treatment to stop significant bleeding through splenic artery embolization or splenectomy is warranted and may be lifesaving.

## Background

In prior studies, the most common etiology of spontaneous splenic rupture was due to hematologic malignancies. In one systematic review, in only 9.1 % of patients were spontaneous splenic ruptures associated with drug and treatment-related causes and these included the use of anticoagulants, granulocyte-colony stimulating factors, thrombolytics, and dialysis [1]. In a more recent retrospective study, 33 % of cases of spontaneous splenic rupture were associated with anticoagulant-antiaggregant use [2]. Spontaneous splenic rupture associated with anticoagulant or thrombolytic therapy, although rare, has been well documented and may be more frequent than initially believed [2, 3]. Despite this, spontaneous splenic rupture associated with novel anticoagulants such as factor Xa inhibitors remains a relatively new occurrence [4, 5]. To date, there have been no published cases of spontaneous splenic rupture that have been found to be associated with the factor Xa inhibitor, apixaban.

Apixaban is a direct and competitive inhibitor of factor Xa indicated for the prevention of stroke in patients with a history of non-valvular atrial fibrillation, prevention of thromboembolism for those who have undergone recent knee or hip replacement surgery, and treatment of acute deep venous thrombosis/pulmonary embolism in appropriate patients [6, 7]. Here we present a case of spontaneous splenic rupture in an 83-year-old man who was on apixaban for stroke prophylaxis due to chronic atrial fibrillation.

## Case presentation

An 83-year-old white man presented to our emergency department with shortness of breath. His past medical history was significant for chronic obstructive pulmonary disease, atrial fibrillation, congestive heart failure, anemia of chronic disease, sick sinus syndrome, pulmonary hypertension, and chronic kidney disease. His medications included probenecid, finasteride, albuterol-ipratropium bromide by inhalation, furosemide, carvedilol, omeprazole, tamsulosin, mirtazapine, ferrous sulfate, metolazone, losartan, budesonide, and prednisone. He was also on apixaban 2.5 mg twice daily for anticoagulation due to non-valvular atrial fibrillation. He did not have a history of bleeding or clotting disorders, although he had presented to our emergency department with complaints of hemoptysis at least once previously due to his cardiopulmonary disease.

**Fig. 1:**
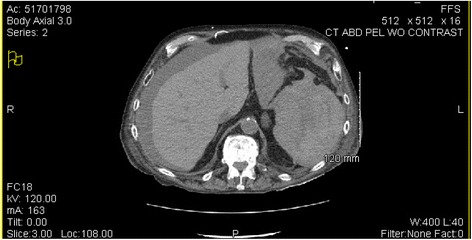
Computed tomography of the patient’s abdomen with splenic rupture and evidence of intra-abdominal bleeding

Acute-on-chronic congestive heart failure and exacerbation of chronic obstructive pulmonary disease were diagnosed. Five days into his hospital stay he awoke with diffuse abdominal pain. He and his wife both denied history of recent abdominal or chest trauma, including falls. After the onset of his abdominal pain, while sitting up in bed, he had a witnessed syncopal event and became unresponsive. A medical emergency response team was called to his bedside and laboratory tests were immediately performed. He recovered and was stabilized, but soon after he developed abdominal distention, tympanic bowel sounds, and altered mental status. He decompensated rapidly with a drop in blood pressure from baseline values of 100/50 mm Hg to 54/32 mm Hg. An immediate abdominal computed tomography (CT) scan was completed at that time with the differential diagnosis being considered including bleeding gastric or duodenal ulcer, ischemic bowel, myocardial infarction, or septic shock. His CT showed a splenic hematoma with associated enlargement of his spleen measuring 13.6×11.5×15.5 cm and blood in his abdominal cavity (Fig. 1). On hematologic tests, his serum hemoglobin was reduced from his baseline value of 9.3 g/dL on admission to 5.3 g/dL (normal range 13.5 to 18 g/dL). His activated partial thromboplastin time (aPTT) was within normal range; however, his prothrombin time (PT) was elevated at 16.1 seconds (normal range, 9.5 to 12.7 seconds) consistent with his use of apixaban.

Rapid transfusion of two units of packed red blood cells, followed by three units of fresh frozen plasma was initiated. Despite attempts at resuscitation, he remained hypotensive and in shock. Due to multiple comorbidities which could potentially complicate surgical intervention, he was urgently transferred to interventional radiology for an emergency splenic artery embolization. An angiogram completed during the procedure showed a splenic artery that was patent and tortuous, with medial displacement of his spleen by a hematoma consistent with what was seen on CT imaging. He was successfully stabilized by embolization of the bleeding source; he was subsequently transferred to our intensive care unit.

The following morning, subsequent to the embolization of the bleeding source, his hemoglobin had decreased from 8.6 g/dL to 6.6 g/dL, which raised a concern that he had recurrent bleeding; a laparotomy was performed with a splenectomy and evacuation of a large intra-abdominal hematoma. During the procedure, 3600 mL of blood and clots were evacuated from his abdominal cavity.

Following the surgery, he underwent prolonged mechanical ventilation due to respiratory failure and temporary hemodialysis for acute-on-chronic kidney injury. He was eventually discharged to a short-term rehabilitation facility for physical therapy after an extensive hospitalization.

## Discussion

Spontaneous splenic rupture is a rare event in the health care setting. The literature has cited hematological, infectious, and neoplastic disorders as common causes of spontaneous splenic ruptures [1, 8]. Medical procedures such as colonoscopy, endoscopic retrograde cholangiopancreatography, and hysterectomy have also been frequent etiologies of spontaneous splenic rupture [8]. In addition, splenomegaly, as was seen in our patient, was a common feature in many reported cases, affecting approximately 55 % of patients in one systematic review [1].

As stated previously in the Background section, anticoagulant therapies continue to be linked to instances of spontaneous splenic rupture. Several reports have described spontaneous splenic ruptures in relation to anticoagulant therapy with heparin and its derivatives [9–11]. Anti-fibrinolytic therapy with tissue plasminogen activator and streptokinase has also been associated with spontaneous splenic rupture [12, 13]. While the pathophysiology for spontaneous splenic rupture remains largely unknown in many instances, one theory states that a spleen with previous microtrauma may rupture when placed under different hematic conditions as is seen in anticoagulant, thrombolytic, or anti-fibrinolytic therapies [14].

Previous data have cited abdominal pain and hypotension as the most common features seen in patients presenting with an acute episode of spontaneous splenic rupture, with approximately 50 % of described patients presenting with abdominal pain referred to their left shoulder [3, 15]. One recent study cited 100 % of patients presenting with spontaneous splenic rupture as having a predominant complaint of abdominal pain [2]. Other studies showed 95 % of patients presented with abdominal pain, 66 % with hypotension, and some with features of hemorrhagic shock [14, 16]. Due to common symptoms, spontaneous splenic rupture may initially be confused with a cardiac etiology or suspected perforated peptic ulcer. Without a history of trauma pointing to the possibility of a ruptured spleen, treatment is often delayed, particularly in patients with a history of cardiac or gastrointestinal issues [3, 14]. Due to the strong correlation between abdominal pain and splenic rupture, contemplation of this diagnosis is warranted in any patient who is on anticoagulation presenting with abdominal pain.

In general, the recommended treatment for splenic rupture includes emergent laparotomy for those who are hemodynamically unstable. As in our case, 17 % of conservatively treated patients underwent secondary splenectomy because of re-bleeding and progressive hemodynamic instability [1]. For hemodynamically stable patients without peritonitis or signs of active hemorrhage on CT, close observation is acceptable [17]. In the future, reversal agents for factor Xa inhibitors may be useful for treatment of this disorder.

## Conclusions

This clinical case demonstrates the importance of prompt recognition of a spontaneous splenic rupture in a patient on anticoagulation with apixaban. Consideration of this diagnosis should be entertained in any patient with abdominal pain who is on anticoagulation, even if there is no history of trauma. Close observation may be done for those patients who are hemodynamically stable, but more invasive treatment should be available and considered if the patient’s condition deteriorates.

## Abbreviations

CT, computed tomography
